# On the generation of internal waves by river plumes in subcritical initial conditions

**DOI:** 10.1038/s41598-021-81464-5

**Published:** 2021-01-21

**Authors:** R. Mendes, J. C. B. da Silva, J. M. Magalhaes, B. St-Denis, D. Bourgault, J. Pinto, J. M. Dias

**Affiliations:** 1 CIIMAR - Interdisciplinary Centre of Marine and Environmental Research, University of Porto, Matosinhos, Portugal; 2 CESAM - Centre for Environmental and Marine Studies, Physics Department, University of Aveiro, Campus de Santiago, 3810-193 Aveiro, Portugal; 3 Department of Geoscience, Environment and Spatial Planning (DGAOT), Faculty of Sciences, University of Porto, Rua Do Campo Alegre, 687, 4169-007 Porto, Portugal; 4 Institut de Sciences de La Mer de Rimouski, Université du Québec À Rimouski, 310 allée des Ursulines, Rimouski, QC G5L 3A1 Canada; 5 LSTS - Underwater Systems and Technology Laboratory, Department of Electrical and Computer Engineering, School of Engineering University of Porto, University of Porto, 4200-465 Porto, Portugal; 6 Instituto de Ciências da Terra, Polo Porto, Universidade do Porto, Rua do Campo Alegre 687, 4169-007 Porto, Portugal

**Keywords:** Physical oceanography, Ocean sciences, Engineering

## Abstract

Internal waves (IWs) in the ocean span across a wide range of time and spatial scales and are now acknowledged as important sources of turbulence and mixing, with the largest observations having 200 m in amplitude and vertical velocities close to 0.5 m s^−1^. Their origin is mostly tidal, but an increasing number of non-tidal generation mechanisms have also been observed. For instance, river plumes provide horizontally propagating density fronts, which were observed to generate IWs when transitioning from supercritical to subcritical flow. In this study, satellite imagery and autonomous underwater measurements are combined with numerical modeling to investigate IW generation from an initial subcritical density front originating at the Douro River plume (western Iberian coast). These unprecedented results may have important implications in near-shore dynamics since that suggest that rivers of moderate flow may play an important role in IW generation between fresh riverine and coastal waters.

## Introduction

Ocean dynamical processes are largely controlled by the fact that seawater is vertically and stably stratified in density, itself determined by temperature, salinity and pressure. The perturbation of this density stratification provides internal pressure gradients that give rise to a number of complex and intriguing phenomena that would otherwise not exist if ocean properties were homogeneous. In particular, density gradients and gravity provide the baroclinic pressure gradient forces that drive internal wave motions in the ocean, whose properties comprise a wide spectrum of time and spatial scales—with periods ranging from minutes (buoyancy period) to hours (inertial period) and wavelengths from a few tens of meters for high-frequency internal waves (IWs) to tens of kilometers for internal tides^1^. These internal motions are generally denominated internal gravity waves or just internal waves and are now widely acknowledged to be frequent features in the global ocean^2,3^.

Nonlinear IWs are particularly interesting when considering observations in a theoretical or modeling framework since these nonlinear waveforms have the ability to retain their form over an extended time period as they propagate energy and momentum away along the background density stratification at basin scales^3–5^ (particularly referring to internal tidal waves, but also valid for internal solitons in deep ocean). This remarkable coherence of form is explained by the balance between nonlinear steepening effects and nonhydrostatic dispersion. An extensively studied equation has been the Korteweg-de Vries (KdV) equation or some variant of it. This equation has been used by Osborne and Burch^6^ to describe internal solitary waves (ISWs) observed in the Adaman sea at moderate depths (approximately 1300 m). The KdV equation is often the first order theory applied when studying nonlinear IWs in the ocean^7,8^.

Most papers discussing nonlinear IWs report that they propagate at the pycnocline, which is typically located between a few meters to tens of meters deep. The sharp density step provides a natural waveguide for high-frequency waves whose energy is trapped in this interfacial-like layer. IWs propagating along the pycnocline may produce a measurable signature in sea surface roughness (formally quantified by means of the *mean square slopes*), making them readily detectable in satellite imagery. This is essentially a result of the current fields within the IWs, which produce convergent and divergent zones (in the form of coherent bands) on the surface that move in phase with the waves below (i.e. subsurface crests and troughs). Introduced by Apel et al*.*^9^, the study of ISWs using satellite images has been successfully applied in the last 40 years. Sensors on-board satellite platforms may provide quasi-daily snapshots with increased spatial resolutions of tens of meters, which, when combined with numerical models and in situ observations, often provide evidences and valuable insights for understanding IWs generation, propagation and dissipation mechanisms^10^. Synthetic Aperture Radars (SARs) are the most used satellite sensors for ISW studies from space, especially since they excel at capturing changes in sea surface roughness^11^. Nevertheless, satellite images acquired in the optical wavelengths of the electromagnetic spectrum, are also very good sources for detecting and studying IWs^26^. These can reveal the coherent crests and troughs of IWs either in the area of direct specular reflection of sunlight from the water surface, or by color signatures originating from different turbidity, chlorophyll concentrations or water constituents integrated in the water column as deep as the satellite effectively “sees” (e.g. da Silva et al.^12^ and Kim et al.^13^).

The generation of ISWs in the ocean is complex, and despite the fact that 45 years of continuous research has passed since the debate Lee and Beardsley^14^ and Maxworthy's^15^ generation mechanisms were put forward, it is still a subject of active research (see section 1 in da Silva et al.^16^ for a detailed discussion). Some of the most common generation mechanisms of IWs are associated with stratified strong tidal flows over large-amplitude sills or submarine ridges, which generate ISWs close to the area of topographic interaction. In these cases, the ISWs often take form of ISW trains, or internal undular bores, and may arise from the relaxation of internal hydraulic (supercritical) flows^15,17–22^, the release of internal lee waves^23,24^, or some form of upstream influence^16^—just to name a few tidal generation mechanisms. Non-tidal generation mechanisms have also been found to be at work in the ocean. For instance, IWs have been found to originate from river plumes^26^, collapsing mixed layers^25^, and frontally forced intrusions. However, in the ocean, the generation of IWs by fronts or buoyant plumes, such as river plumes, have not been extensively studied, and observations are scarce. Note that the IWs reported in Bourgault et al*.*^26^ are similar to those generated by the Columbia River plume^27^, with the difference that the former does not require the flow to be initially supercritical, contrary to the latter.

In this paper, we explore the possibility of IWs generation by an essentially subcritical river plume front off the Portuguese coast (Douro River), noting that the evidence and explanations given here may occur in many other coastal zones of the world, and hence highlighting a generation mechanism that has been overlooked.

## Results

### Satellite observations

**Figure 1:**
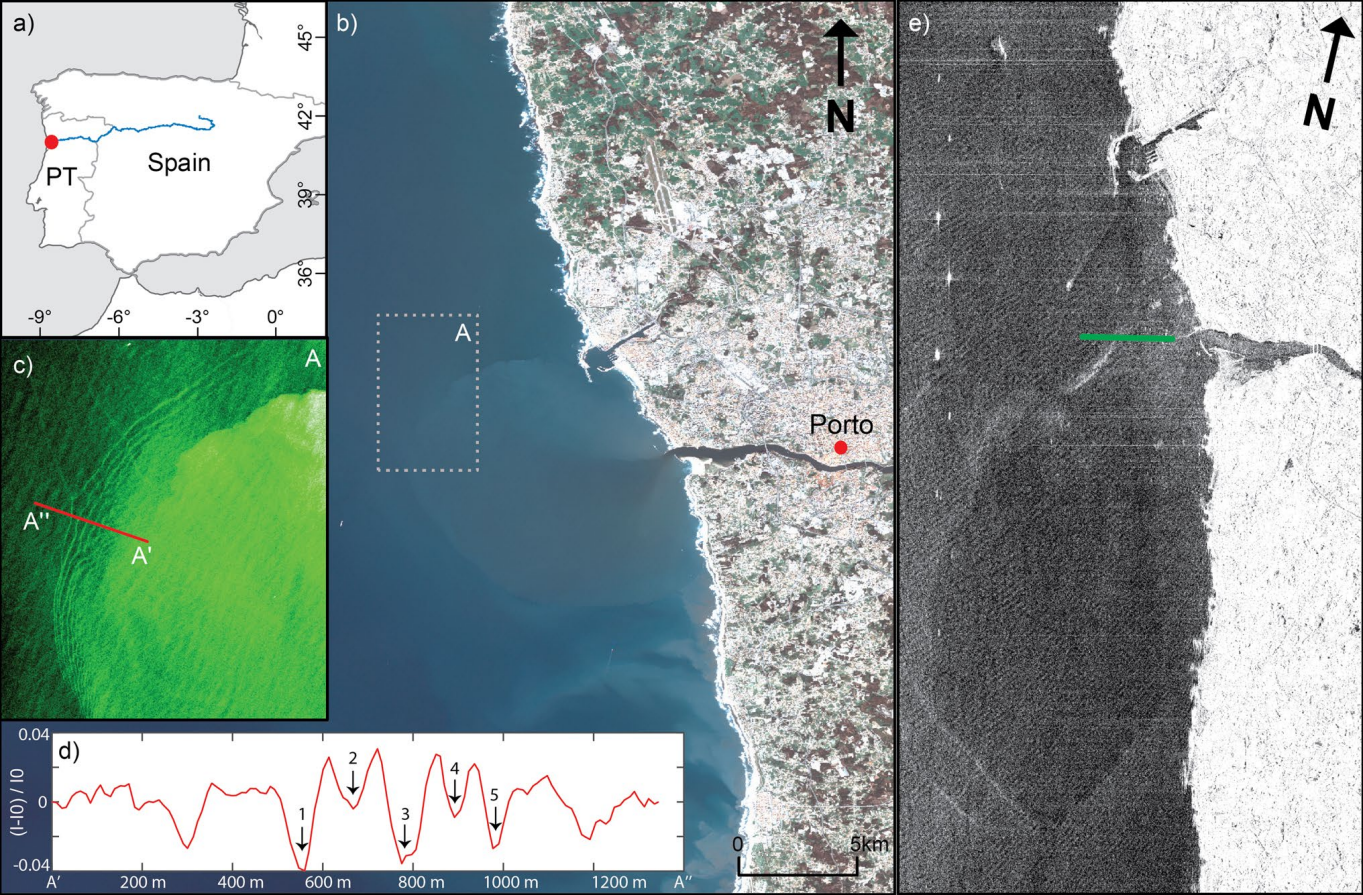
Map of the study area off Douro River; (**b**) Douro River Plume (Sentinel 2A 16 Mar 2017); (**c**) IW generation at the plume front (green-channel); (**d**) Transect corresponding to the red line in (**c**) with evidence of 5 internal solitary waves between A′ and A″; (**e**) TerraSAR-X (German remote sensing synthetic aperture radar) image acquired on April 23, 2017. The reference AUV tracks (data showed in Fig. 2) are demonstrated by the green line. Image (**b**,**c**) processed using SNAP v6.0 (http://step.esa.int/main/download/snap-download/). Data of the image (**d**) were obtained through SNAP v6.0 and processed using Matlab (R2017a, https://www.mathworks.com/products/new_products/release2017a.html). Image (**e**) was processed using Matlab (R2017a). The figure composition and the image (**e**) were created in ArcGIS v10.5 (https://desktop.arcgis.com/en/quick-start-guides/10.5).

The new Sentinel-2 ESA mission has been revealing small-scale surface expressions such as IWs generated by river outflows. Although it is a land-oriented mission, the high-spatial-resolution (10 m) on the visible range wavelengths allows the study of small-scale features in the coastal regions^28^. The present study was initially motivated by satellite observations of coastal IWs manifestations off the Douro River plume (southwest coast of Europe, Portugal), traveling seaward (Fig. 1). Alternating bands of brighter and darker strips, coherently organized as one wave or wave packets, are observed in 36 images. These features can be interpreted as the surface signatures of IWs propagating at the interface between the upper and the lower layer^12,29,30^. Considerable IWs activity has also been detected farther north and south around the plume region at 30 m depth on average (25–40 m), although the largest number of observations (21 out of 27 wave packets) was located SW of the mouth, in the average direction of the Douro plume propagation^31^. Sentinel images with evidence of the IW generated from the plume are also not restricted to any particular season. Even though, the majority were found in summer (15 out of 36) and winter seasons (11 out of 36).

A total of 27 different wave packets and 111 individual IWs were identified in the 18 images analyzed in detail (see “Methods” section for more details). The average of the crestlength for each wave is about 2.8 km, however, its distribution is strongly skewed towards the lower end, with values ranging from 500 m up to more than 15 km. Wavelength values range from 20 m to a maximum of about 141 m. The dataset has a mean value of 60 m and 90% of the observations have a characteristic wavelength, *λ*, between 40 and 94 m (Fig. 1d). These values are typically much smaller than those found by Stashchuk and Vlasenko^32^ (200 m) and Pan et al*.*^33^ (600 m–1.2 km) for IWs generated in the Columbia River plume and higher when compared with values found for IWs in the supercritical plumes from small mountainous rivers in Russia (30–60 m—Osadchiev^34^). As expected, a comparison with horizontal length scales of oceanic IWs in the Portuguese continental shelf near Porto, da Silva et al*.*^35,36^ encountered larger values, with a difference of about 1 order of magnitude.

The combination of satellite data, wind, tide, and river discharge in situ data also provided an opportunity to examine the dependence between the IWs generation and typical plume driver mechanisms. The identification and location of waves in satellite imagery suggested a relation with tides. The great majority of waves possibly generated by the plume were observed after low-tide (flood or slack conditions). A trend was observed between the distance of the wave trains to the river mouth to the time after the low-tide peak (see supplementary material SM1), relating the IWs generation with the ebb-tidal pulses, as was found for the Columbia River plume^37^. However, no significant difference was found between spring and neap tidal cycles in terms of number of images (11 and 16), spatial distribution and energy proxy ($E^{*}$ = 0.38 m and $E^{*}$ = 51 m) of IWs trains (see “Methods” section).

IWs were rarely observed in Sentinel imagery when the river discharge was smaller than the 25th percentile (< 144 m^3^ s^−1^) or higher than the 75th percentile (> 549 m^3^ s^−1^). Only 4 wave packets were identified in those conditions, the majority of IW manifestations was observed during moderate river discharge. The majority of the images were found under calm (< 3 m s^−1^) to moderate (3–6 m s^−1^) wind conditions. This was somewhat expected, as the stratification near the coast will be weakened or disappeared due to the strong mixing by a high river discharge or strong winds. In these conditions, the probability of generating IWs decreases. Therefore, the upper-layer density stratification becomes essential for this mechanism.

### Cross-front observations

**Figure 2:**
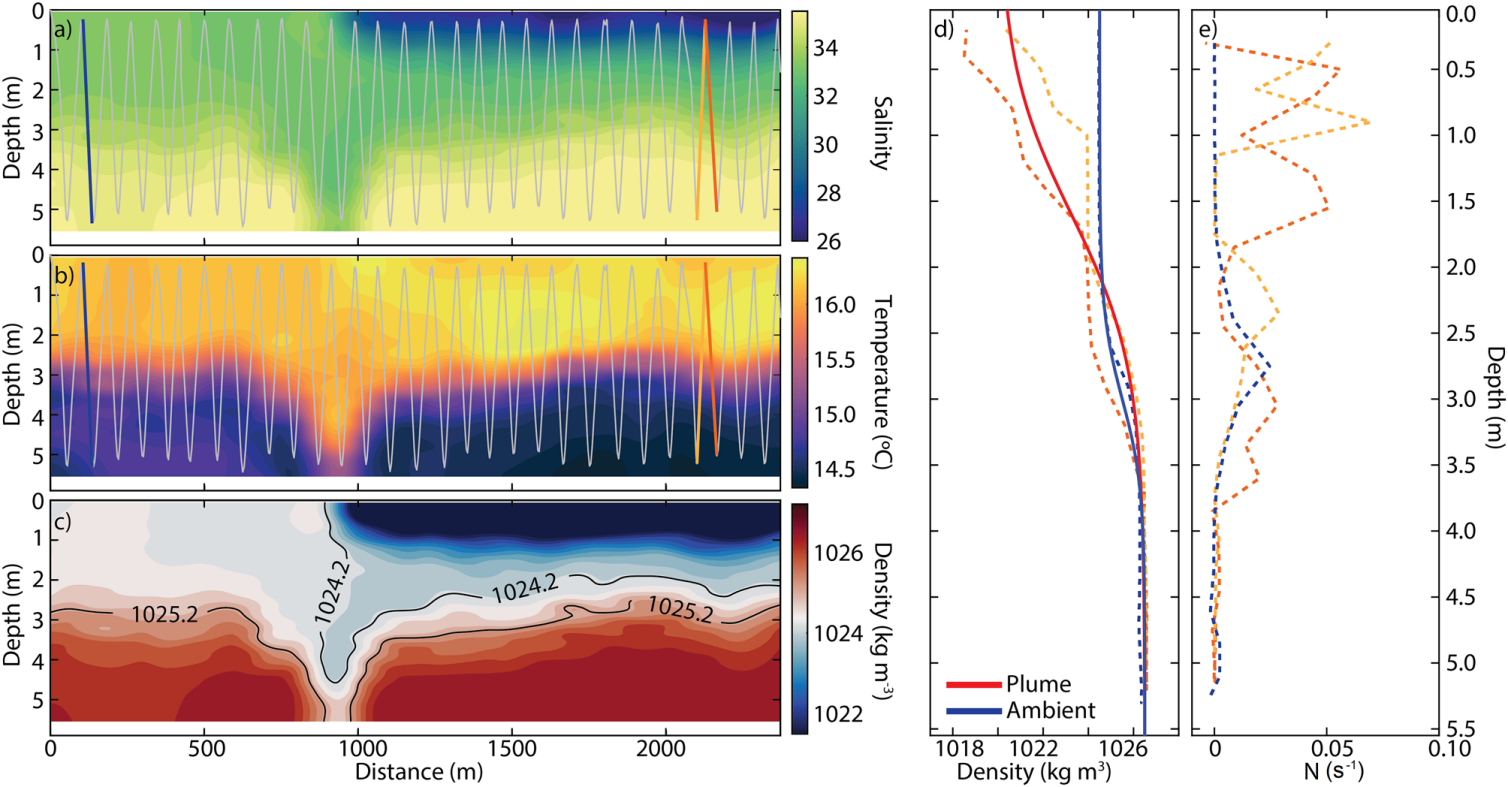
AUV observations and background conditions. AUV cross-front transect of salinity (**a**) and temperature (**b**) with the corresponding yoyo trajectories (grey line). AUV-based density contour map (**c**). Observed (dashed) and idealized (solid) background density (**a**) and Brunt–Väisälä frequency ($N$) profiles on the ambient coastal ocean (blue) and plume (red) side. Dashed dark and light orange lines correspond to upward and downward trajectories, respectively, completed by the AUV in the plume side.

The coastal stratification off Douro River is driven by water temperature in early spring and summer, however low salinity water from river estuarine outflow can strengthen the stratification, especially during late fall, winter, and early spring. Several field deployments using AUVs synchronized with the tide, and Sentinel 2 and/or TerraSAR-X overpassing were performed nearby the Douro River plume during these periods in 2017 and 2018. IWs generated by the plume are not frequent and, unfortunately, we could not match any in situ observations and satellite imagery with any indication of IWs. Between late winter and early summer of 2017, IWs propagating seaward were identified in six Sentinel-2 images, likely generated by the plume. Figure 1b exemplifies this by showing stripe patterns associated with a train of NLIWs that radiate away from a turbid plume front 15 min after the time of low water (11:15 UTC). In our study, the largest contrasts are observed in the image profile (Fig. 1d) using a single-band analysis on the green channel of Sentinel 2, in agreement with previous studies^31,38,39^. The synthesis of cross-front water temperature and salinity data obtained during an AUV mission, shown in Fig. 2, elucidates more of the vertical and horizontal structure of the density field. Despite the fact that these observations were obtained about 1 month after the Sentinel 2 acquisition (Fig. 2b), the meteo-oceanographic spring conditions were nearly identical and comparable. The time of acquisition corresponds to a low-water period with identical sea surface height (0.8 m—16 Mar; 0.9 m—23 Apr) and tidal range (2.4 m; 2.2 m). The river discharges (289 and 135 m^3^ s^−1^) were both below the average (549 m^3^ s^−1^), and wave heights were between slight to moderate (0.5–1.5 m) in both situations. The weather conditions were typical for the season in the region. Both days were sunny (18–21 °C max air temperature) with a light breeze (1–3.5 m s^−1^) blowing from NW. It is reasonable, albeit speculative, to assume that the coastal environment and plume stratification were similar in these two days, and correspond to typical spring conditions for this coastal region.

Plume and background water stratification are summarized through a cross-frontal transect in the Douro River plume obtained by AUV on April 23, 2017 (Fig. 1e). The water column is shown to be stratified in both water temperature and salinity (Fig. 2). On the plume side, a strong low-salinity signal is observed (26–28) near-surface (0–1.5 m) (Fig. 2a). The riverine influence is also noted in the background coastal water (left side). Here, salinity values from 30 and 34 indicate that this water is possibly related to a reminiscent plume from the previous ebb^40^. The water below 3 m on both sides presents a more typical oceanic origin (S > 34). A thermocline is observed at 3 m depth along all the transect with water temperature differences of about 1.5 °C °between near-surface (~ 16 °C) and below the average thermocline depth (~ 14.5 °C) (Fig. 2b). Therefore, the persistent thermocline is mostly explained by sunlight heating and the lack of active mixing drivers, such as wind and waves. However, the difference between background and plume waters in terms of water temperature is more subtle. The near-surface temperature is about 16.5 °C, which is associated with the warmer estuarine waters (Fig. 2b).

Figure 2c shows the associated density field that clearly reveals the river plume that appears as a tongue of light surface water. The figure also shows the stratification on the shelf that determines the conditions of IW radiation^32,41^. An important feature of these observations is seen in the convergence region associated with the front where the prominent pycnocline is depressed (> 2.8 m) right ahead of the front, which appears as a bore-like structure (head of the plume). Such a bore can evolve into a packet of IWs^27^. This bore was not observed in the subsequent transect 20 min later. We hypothesize that it had evolved into a packet of IWs that propagated away from the front region, too far to be sampled. Nevertheless, the second transect allows us to estimate the maximum front velocity ($u_{2}$) by 0.14 m s^−1^, slightly smaller, but in the same order of magnitude as those found in the literature for other river plumes^27,42^.

### Numerical simulations

We carried out numerical simulations to determine whether the hypothesis of the generation of IWs by the propagating plume from the Douro River was possible in the hydrographic conditions of 23^rd^ April. Also, we analyzed the numerical results to see whether they could provide an explanation for the existence of IWs surface signatures seen on satellite imagery, matching with the derived length scales from other events, namely 16th March 2017.

**Figure 3:**
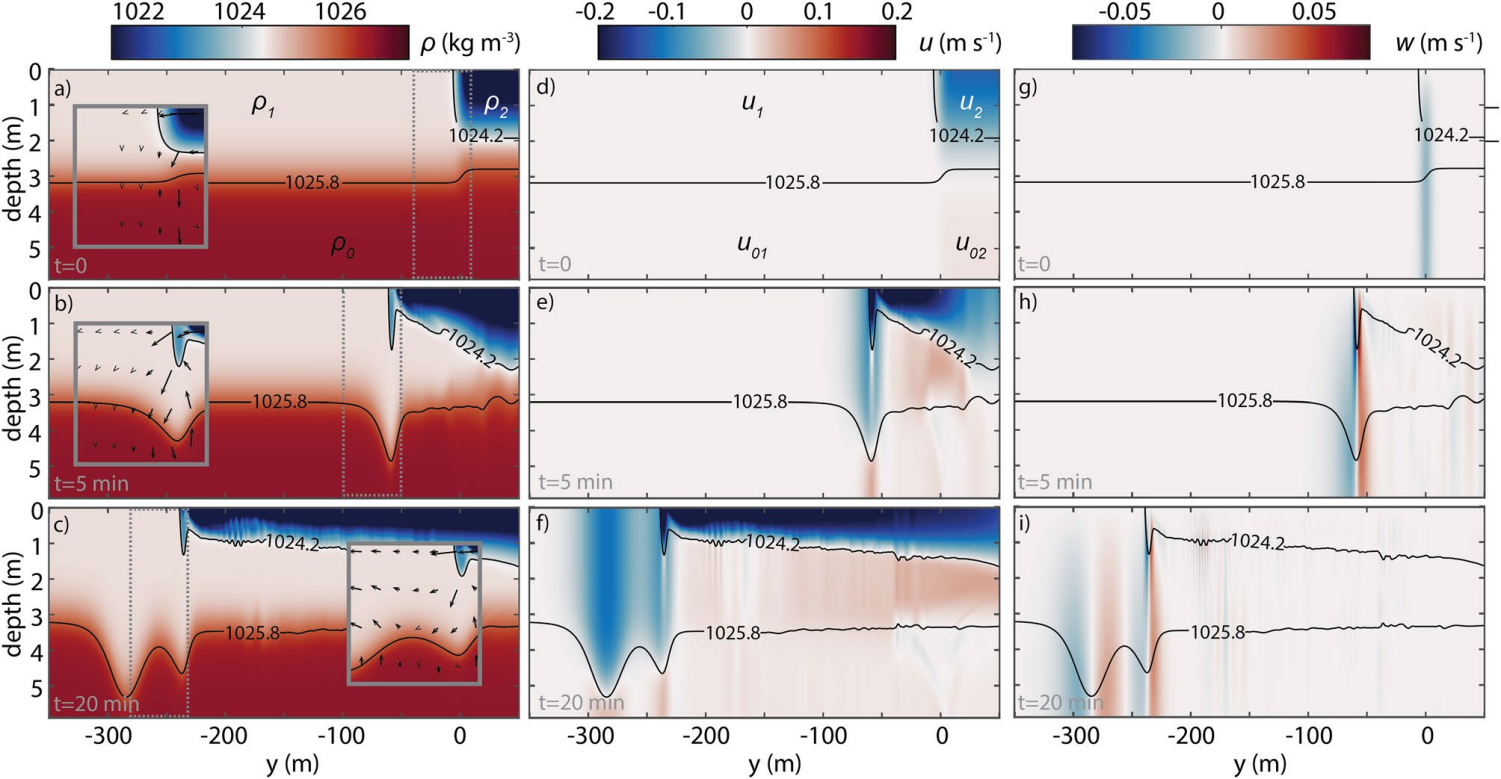
Numerical results. Numerical simulations of internal solitary waves generated by a subcritical plume front. (**a**–**c**) Density $\rho$, (**d**–**f**) eastward current $u$ and (**g**–**i**) upward current $w$ with reference density contours superimposed as grey lines. The top row (**a**,**d**,**g**) represents the model initialization ($t = 0$) with parameters $\rho_{0}$, $\rho_{1}$, $\rho_{2}$, $u_{0}$, $u_{1}$,$u_{2}$, $u_{01}$ and $u_{02}$ as defined in the “Methods” section and given in Table 1. The middle row represents the moment of the first front collapse with the generation of the first pycnocline’s perturbation below ($t = 5 \,{\text{min}}$) and the bottom panel represents an entire soliton progression ($t = 20\,{\text{min}}$). For the sake of simplicity, only results from run #1 are shown. The inset on density panels (**a**–**c**) highlights the front evolution within the dashed boxes. Note that not all the velocity vectors are shown and $w$ is increased by a factor of 5 for better picture legibility. Only the top 6 m are shown but the model domain is 30 m deep.

Our analysis follows the methodology and uses the same model approach as Bourgault et al*.*^26^, considering the nonlinear and nonhydrostatic processes involved (see “Methods” section for details). All simulations were carried out in a 30 m ($H$) deep flat-bottom open channel, which is the typical depth of the region where wave trains were identified in satellite imagery (Fig. 1). The model was initialized with a three-layer idealization of the plume front and ambient water (Fig. 3a,d,g;) based on observations (Fig. 2). The usual type of operation of AUVs or glider CTD sensors in water-column measurements often resulted in a thermal-lag between up and down trajectories^43^. The phenomenon is observed in Fig. 2b, in the plume side particularly. The up trajectory curves appear bowed upward and the down trajectory curves appear bowed downward. Due to the thermal-lag problem, the water temperature tends to increase with depth, so a slight positive salinity (and density) error occurs on the up trajectory going from warm (ambient) to cold (plume) water and a negative error occurs on the down trajectory going from cold to warm water^43^. Consequently, we opted to choose a simplified density profile to initialize the model based on observations from both up and down trajectories in the plume side (Fig. 2—see “Methods” section) to minimize potential fault assumptions.

The initial state in the control run (run #1) is characterized by a convergent flow at the plume layer ($u_{2} = 0.14$ m s^−1^) with an ambient and plume sheared environment. All runs correspond initially to non-equilibrium cases ($\varepsilon = 0.23$) and interfaces are dynamically stable with minimum interfacial shear Richardson number $Ri_{P} = 6.57$ and $Ri_{A} = \infty$. The river plume region is initially subcritical with a corresponding composite Froude number of $Fr_{P} = 0.44$ for the control run with higher plume speed.

Figure 3 illustrates density and velocity ($u$ and $w$) cross-front section at 3 different stages of the plume evolution. The plume head is well developed after 5 min and continues to grow with a rotational movement, which can be seen as the initial stage of the bore evolution. The full detachment of the plume head in the surface was not observed, however, the continuous growing bore compresses the pycnocline below, as a kind of piston, generating IWs in front of it (Fig. 3b). The leading wave starts to separate from the main body of the plume from this moment and 15 min later it has detached from the plume (Fig. 3c). After that, continuous waves are formed starting to evolve into a series of rank-ordered IWs. The leading solitary wave on run #1 has an amplitude of about $a$ = 2.11 m and a maximum horizontal and vertical velocities of $u$≈ ± 0.13 m s^−1^ and $w$≈ ± 0.04 m s^−1^ (Fig. 3f,i), propagating offshore along the pycnocline at 3 m depth in the ambient coastal water, where the primary mode and thus fastest possible linear wave speed is $c_{0}$ = 0.23 m s^−1^. Moreover, such wave can be considered as nonlinear ($a/h_{1} \approx 1$) and long relative to the surface layer thickness ($\lambda /h_{1}$ = 24). These numerical results indicate that such a solitary wave in a stratified and sheared environment has a horizontal length scale of about $\lambda$ = 45 m, which corresponds to 75% of the average and within the range of dimensions found in satellite observation on March 16, 2017 ($\lambda \approx$ 75 m—see “Satellite observations” section). The IW amplitude derived from satellite observation, $a_{sat}$, is found to be 0.21 m, considering the solitary wave half-width $D \approx 55$ m, which is the median value of all individual waves detected in the green intensity profile (Fig. 1d). That result compared well with 0.65 m, the IW amplitude found in the numerical simulations when the initial motion is zero. That result should be taken cautiously because of the model limitations as well as the uncertainties associated with the evaluation of $D$ in the Sentinel-2 imagery due to the nominal pixel resolution of 10 m can play. A small variation in the quantification of the value $D$ will lead to a significant difference in $a_{sat}$.

**Figure 4:**
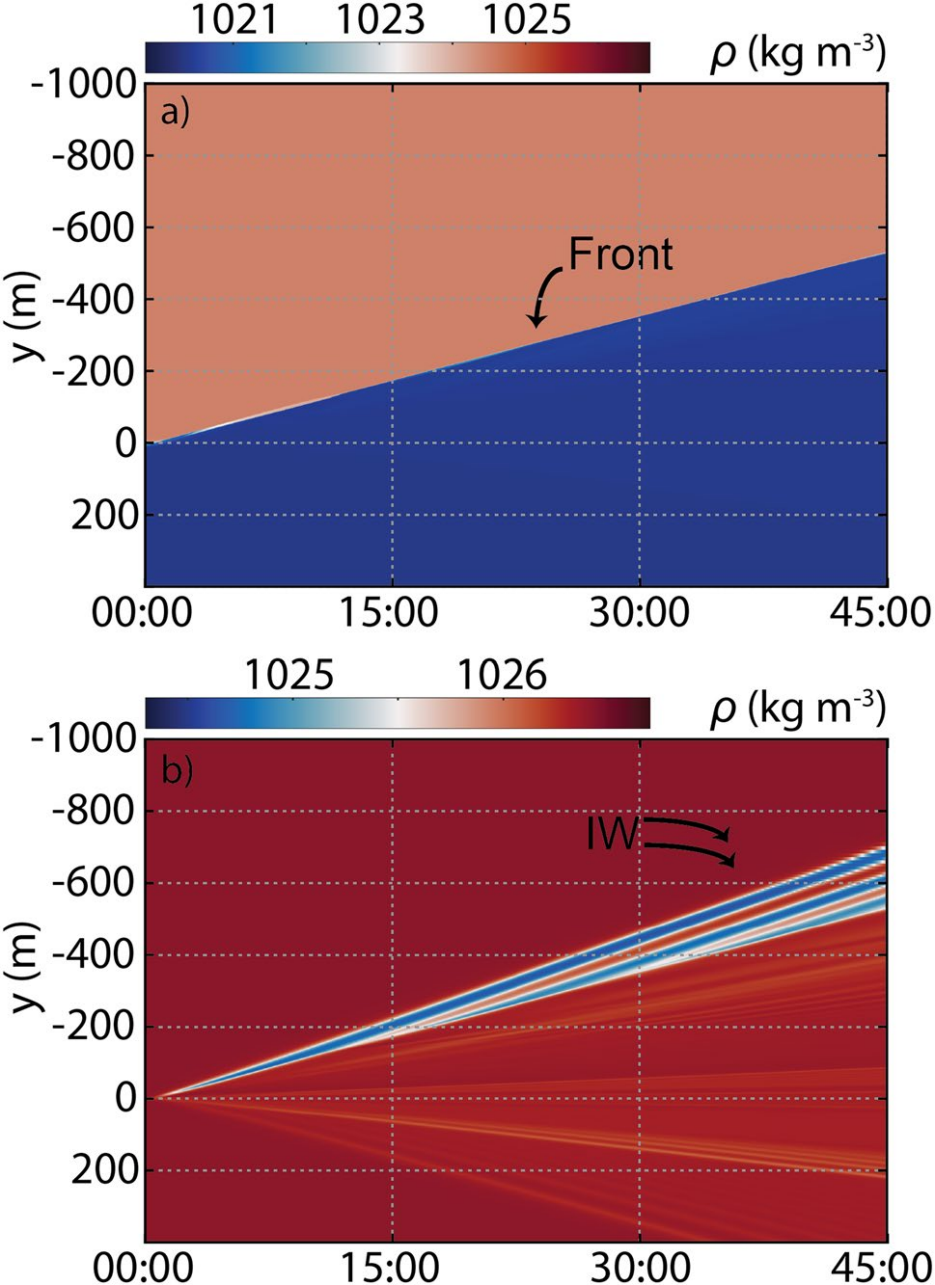
Plume front and IW velocities. Hovmöller diagram generated from model results from run #1 at the surface to highlight the front progression (**a**), and at 3.5 m depth to focus on internal solitary wave’s progression (**b**).

Density sections shown in Fig. 3 represent well the main stages of wave evolution described by Nash and Moum^27^ for the Columbia River plume. The growth of the plume head, the generation of the IWs as pycnocline depression ($a$ < 0, because $h_{1} < h_{2}$) ahead of the front and the wave packet detachment and the consequent free propagation are identified, although, here, wave generation does not require the buoyant flow to be initially supercritical. The leading edge of the plume is visible in the Hovmöller diagram depicted in Fig. 4a as a linear feature at the surface layer. It is shown to propagate a distance of approximately 180 m in 15 min, resulting in a velocity of about 0.2 m s^−1^ ($u_{f}$, Table 1). The comparison, therefore, demonstrates that the plume front propagation speed is less than the first mode linear phase speed of the IW, $c_{0}$ = 0.23 m s^−1^, that is, the plume is subcritical. The characteristic phase speeds for IWs using the model’s continuous stratification^44^ is still consistent with an essentially subcritical river plume and the result ($c_{0}$ = 0.22 m s^−1^) is in practice the same as that found previously for a two-layer model. Three IWs emerge from the front at 3.5 m depth as seen in the Hovmoller diagram of Fig. 4b and these have constant phase speeds given how linear the traces are in this space–time representation. However, the density difference caused by the pycnocline depression at the same depth is found to be more prominent at the leading than in the subsequent waves. This could be interpreted as a consequence of the rank order wave packet formed in which the leading wave has a higher amplitude. All of the waves are faster than the plume front and the leading wave exhibits a velocity of about 0.27 m s^−1^ (about 2 times slower than the IWs generated by Columbia River plume^27^) (Table 2). As expected, this velocity is higher than $c_{0}$, due to its nonlinearity. The Korteweg-de-Vries (KdV) theory is commonly applied to characterize nonlinear IWs in a two-layer system^45^. In this theory, the wave phase speed is given by $c_{KdV} = c_{0} \left( {1 + \left( {a\left| {\left( {H - h_{1} } \right) - h_{1} } \right|} \right)/2h_{1} \left( {H - h_{1} } \right)} \right)$. $c_{KdV}$ result is 0.30 m s^−1^ and, although close to Hovmöller estimation, the wave velocity is overestimated. Despite the not so strong nonlinearity character of this wave ($a/h_{1} < 1$), KdV theory starts to diverge when wave amplitude is up to about 0.4 times the value of $h_{1}$ depth (here, $a = 0.7 h_{1}$) and therefore it is not the best nonlinear model for these moderate nonlinear waves^45^ (Table 2).

**Table 1:** Parameter values (in S.I.) used for each simulation carried out.

#	$\rho_{0}$	$\rho_{1}$	$\rho_{2}$	$u_{01}$	$u_{02}$	$u_{1}$	$u_{2}$	$h_{1}$	$h_{2}$	$d_{1}$	$d_{2}$	$d_{hor}$
1	1026.5	1024.5	1020.0	0	0.008	0	− 0.14	3.0	1.3	0.65	1.15	10
2	1026.5	1024.5	1020.0	0	0.006	0	− 0.1	3.0	1.3	0.65	1.15	10
3	1026.5	1024.5	1020.0	0	0	0	0	3.0	1.3	0.65	1.15	10
4	1026.5	1024.5	1018.0	0	0	0	0	3.0	1.3	0.65	1.15	10
5	1026.5	1024.5	1022.0	0	0	0	0	3.0	1.3	0.65	1.15	10
6	1026.5	1026.0	1020.0	0	0	0	0	3.0	1.3	0.65	1.15	10
7	1026.5	1022.0	1020.0	0	0	0	0	3.0	1.3	0.65	1.15	10

**Table 2:** Summary table (in S.I.) from numerical results.

#	$u_{f}$	$c_{IW}$	$a$	$Ri_{P}$	$\varepsilon$	$Fr_{P}$
1	0.20	0.27	2.11	6.52	0.226	0.44
2	0.18	0.25	1.65	12.78	0.226	0.32
3	0.14	0.23	0.65	∞	0.226	0
4	0.15	0.23	0.75	∞	0.198	0
5	0.05	–	–	∞	− 0.011	0
6	0.19	–	–	∞	0.356	0
7	− 0.03	–	–	∞	− 0.913	0

**Figure 5:**
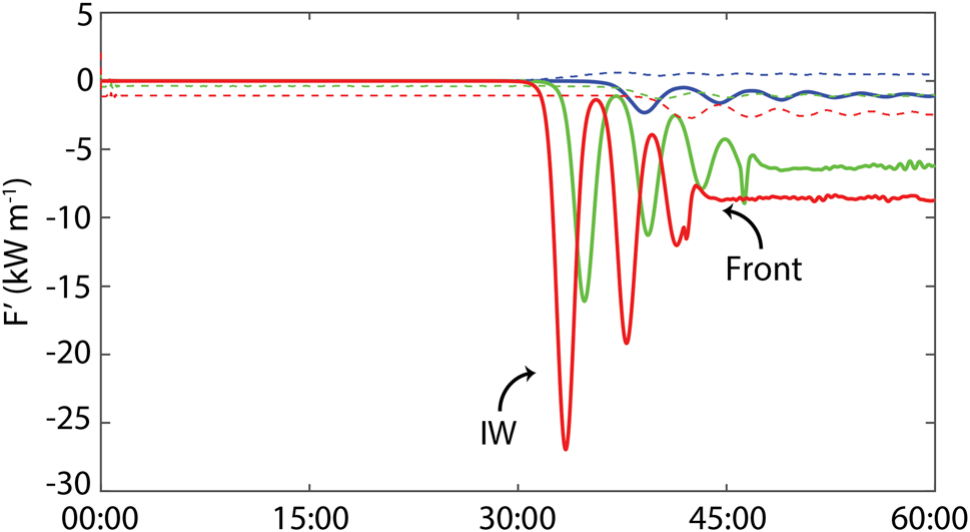
Energy fluxes. Simulated seaward (solid) and landward (dashed) depth-integrated perturbation energy fluxes *F*′ at $x = - 500$ m for different numerical simulations. The curves represent the numerical results using the parameters listed in Table 1. Red, run #1; green, run #2; blue, run #3.

The plume head emits continuously 3 IWs every five buoyancy periods ($\sim$ 3.3 min) until its front reaches the marked distance. The leading wave in run #1 generates a maximum instantaneous depth-integrated energy flux perturbation of 27 kW m^−1^(Fig. 5). Moreover, IW generation in subcritical initial ambients was tested by exploring the sensitivity of the plume velocity (run #2 and run #3) and the stratification in the ambient and plume sides (runs #4 to #7) with no initial motion. Run #2 characterizes the case of a lower river plume speed ($u_{2}$ = 0.10 m s^−1^) and run #3 represents a null plume speed, an extreme situation to simulate the wave emission in the slack of the tide but with identical initial density field. Although all these numerical results show an IW generation at the front and a consequent offshore propagation, the decrease of the plume velocity causes nevertheless a wave nonlinearity decrease. The wave structure separates from the plume 1.5 min and 6 min later (Fig. 4) and the wave velocity decreases to 0.25 m s^−1^ and 0.23 m s^−1^ in runs #2 and #3, respectively (Table 2). The run #3 shows a leading wave velocity equivalent to $c_{0}$, therefore, with weak nonlinearity ($a/h_{1}$ = 0.2) the initial disturbance tends to evolve into small-amplitude ($a$ = 0.65 m) long waves according to linear dispersion (Table 2). However, a small increase (0.1 m s^−1^) in the plume velocity (run #2), leads to an increase of the leading wave amplitude of about 2.5 times. Indeed, numerical results show that waves generated in run #3 are at least 10× and 7× less energetic than run#1, and run#2 (Fig. 5, blue). The sensitivity runs #4 to #7 show that stratification can also play a significant role, altering the IW characteristics and limiting its generation. Run #4 represents a small decrease (2 kg m^−3^) in the plume layer density while the other parameters remain as in Run #3. Although the front velocity increases (0.15 m s^−1^) in run #4, the main patterns among these two simulations can be considered equivalents (Table 2). IWs propagating seaward are detected in run #4, with the same velocity (0.23 m s^−1^) and slightly higher amplitude (0.75 m). In runs #5 to #7, the IW generation at the plume front was not observed. On other hand, in runs #5 and #7, the surface layer of the ambient intrudes into the two-layer plume region (please check the simulations videos in the SM). Here, we observed IWs generated at the head of the intrusion and propagate landward with a significant amplitude in run#7 (1.1 m).

## Discussion

**Figure 6:**
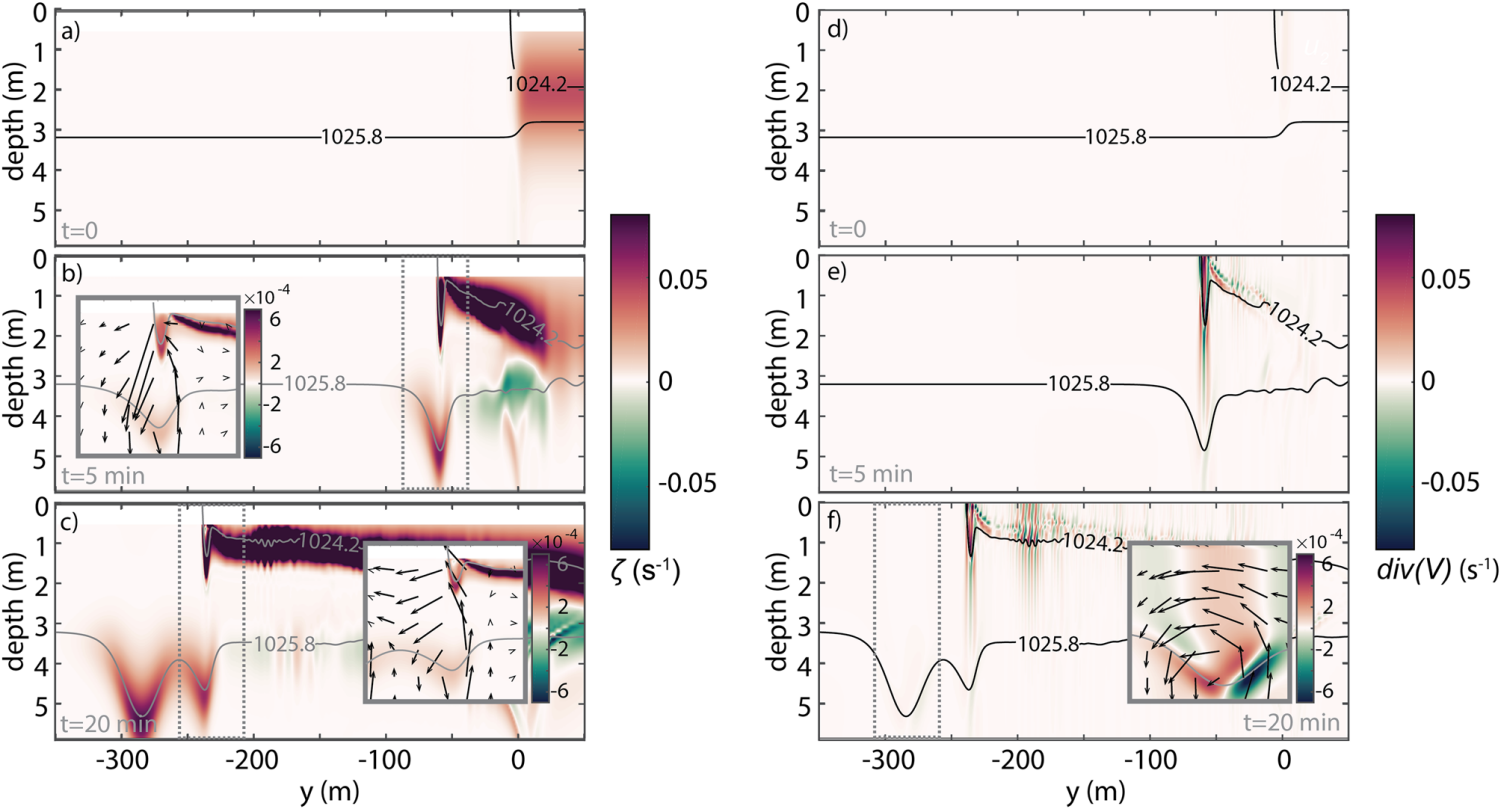
Vorticity and Horizontal Divergence. (**a**–**c**) Vorticity $\zeta$ and (**d**–**f**) horizontal divergence $div\left( V \right)$ with reference density contours superimposed as grey/black lines. The top (**a**,**d**), middle (**b**,**e**), and bottom (**c**,**f**) panels represent the same time-lapses of Fig. 3. The insets on Fig. 5b,c,f highlight the vorticity and velocity field in the head of the front (dashed boxes) and the inset on Fig. 5f shows the horizontal divergence and velocity field in the internal solitary wave’s progression ahead of the plume front (dashed box). Note that not all the velocity vectors are shown and $w$. is increased by a factor of 5 for better picture legibility.

When the plume water collides with the ambient shelf waters, it sinks in the area of the plume leading edge and forms a gravity current head with high positive vorticity (Fig. 6b). The head of the plume never detaches completely from the main buoyancy water body like a trapped core, but it grows in depth and vorticity until the pycnocline perturbation, that was locked to the plume, advances into the coastal water as an IW. This process was already explained by Nash and Moum^27^, however, these authors point out that the transition of the plume flow from supercritical to subcritical is the moment when waves can be released from the front (in Columbia River plume).

A zone of surface flow convergence forms ahead of the river plume, as shown by the velocity vectors (arrows) in Figs. 3b and 6b. As the plume front propagates offshore (to the left in the images), the pressure gradient generated by the lateral density gradient in the background drives a rightward countercurrent opposing the river plume. This creates a zone in front of the plume with a strong downward flow (vorticity). The downward flow, unable to penetrate the pycnocline, splits into two currents that flow horizontally away from the plume in the bottom half of the surface layer. This creates a second core of positive vorticity, representing the beginning of the vertical displacement in the pycnocline, which, henceforth, will progress to the generation of the leading IW. Interestingly, some of the water from the ambient flow returns toward the head of the front while some subducts below the plume and travels toward the right of the domain, as a partial-depth intrusion case^26,42^ (Fig. 6a–c). The resulting currents create strong vertical shear, both behind and ahead of the plume front, which eventually stimulates small-scale instabilities landward (Fig. 5—dashed lines). That current beneath the plume tends to decrease the front slope, which, therefore, attenuates the amplitude of the front head when the IW is released. However, the persistent convergence at the front (Fig. 6d–f) continues to promote the fluid collapse, creating a new pycnocline perturbation and a subsequent second wave generation. This description fits well with the resonance theory of Grimshaw and Smyth^46^ which predicts the generation of IWs by stratified flow over a topographic obstruction, even in subcritical initial conditions^47^. Here, the head of the plume can be considered as a moving topographic feature at surface^32^. Our results also matched well the main conclusions from the numerical simulation of White and Helfrich^48^, which supports our hypothesis that the IWs observed were generated by resonance. Based on their Fig. 14(a), our run #1 is in the limit of the resonant band, assuming $\left( {\rho_{0} - \rho_{1} } \right)/\left( {\rho_{0} - \rho_{2} } \right) \approx 0.3$ and $u_{2} /\sqrt {g^{\prime}H} \approx 0.18$. It is considered a type II regime, exhibiting a resonant generation of nonlinear IWs, whereby the energy is transferred from the front to the waves, which are continuously generated in the front. In the same two-layer conditions but with a decreasing front velocity (Run #2 and Run#3), the results fit better with type I^48^, subcritical regime, in which IWs are generated but with a small amplitude and an intrinsic linear speed close to $c_{0}$ (Table 2)^49,50^.

We also noted a propagation of vortex perturbations with a singular vertical vorticity distribution which yields IW generation^51^. The pycnocline depression that results from the IW passage is identified by the positive patch of vorticity (anticlockwise rotation, Fig. 6c,e). At the surface, a convergence patch is observed ahead of the subsurface vorticity core, while behind the wave center, a velocity divergence is detected. This is the typical description of the IWs velocity structure discussed by, for example, Lamb^52^ and Moum et al*.*^53^. Despite no plume-water detachment was observed at the surface, the convergence and divergence bands are a sufficiently distinctive signature to be identified by different remote sensing imagery (e.g. SAR, OceanColor).

The numerical results combined with satellite observations present evidence that the Douro River plume can be a source of IWs on the Iberian coast. Also, the results indicate that wave generation does not require the plume flow to be initially supercritical as initially hypothesized^24^. While higher front speed conditions favor the generation^27,32,41^, it is not a precondition for generating IWs in a frontally forced coastal water collision. Here, we presented evidence that 2 m amplitude IWs generated by plumes from rivers with moderate discharges can be observed even in subcritical initial conditions and be more common than previously reported. Therefore, the generation process is much more sensitive to ambient water stratification, which was also described in previous studies^32,41^. In the limit of well-mixed waters on the shelf (run #6), IWs will never radiate from the plume. On the contrary, a small stratification increase provides necessary conditions for the collapse of the front and, consequently, for the IWs generation. However, if $\rho_{1}$ is close to $\rho_{2}$, the ambient coastal water at the surface can frontally force interfacial gravity currents intruding into a two-layer and vertically stratified plume ambient. That is observed in simulations (runs #5 and #7), with IWs generated at the head of the intrusion and propagate landward. These numerical results although possible are unlikely to be observed in the Douro Plume because of the coastline proximity, the decrease of depth, and high vertical mixing promoted by tidal-currents and surface wave-breaking.

Although less energetic than large-amplitude waves that propagate through deep-sea, the plume-generated waves can play an important role in biology, sediment resuspension, and turbulent mixing and are sufficiently strong to be observed from space. Since river plumes are regions of high biological activity and organic and inorganic material aggregation^54^, the IWs described above can provide an efficient transport mechanism between the plume and adjacent coastal waters. Relevant implications for bottom resuspension material, biology, and turbulent mixing are expected in the shallow area along the waves’ path^5^.

The increase of spatial and temporal resolution by recent and future satellite missions will bring more insightful information regarding the necessary initial environmental conditions for IWs generated by river plumes and other fronts in the coastal ocean. Also, the use of AUVs measurements for these small-scale coastal processes can be extremely useful to obtain synchronous water column data with higher spatial and temporal resolution.

## Methods

### In situ data

Observations were undertaken using a LAUV (Light Autonomous Underwater Vehicle) on April 23^rd^, 2017 along several longitudinal transects crossing the Douro River Plume front with a vertical yo-yo trajectory (Fig. 3). The LAUVs are rugged, efficient, lightweight vehicles with multiple applications^55^. They provide a robust autonomous approach for ocean data collection, comprising communications, computing and navigation sensors. In this study, the water temperature and salinity data are as a function of depth and position along the transect monitored.

The vertical thermohaline structure was measured from surface to a depth of 6–7 m by a CTD (RBR XR-620CTD) mounted on the AUV nose with a sampling period of 2 s and an averaged vertical resolution of about 0.5 m. The data presented here were collected between 17:42 and 18:51 UTC (Fig. 3). The fieldwork was synchronized with the end of the ebb-tidal cycle (low tide peak at 18:50 UTC) when the plume reaches its larger dispersion, and with Terra-SARX satellite overpassing (18:25 UTC—Fig. 1e).

The AUV dataset was pre-processed on Neptus software^56^ by correcting estimated positions with GPS data acquired at surface, and then, in *Matlab* software, by removing outliers and spurious data. An optimal and smoothing interpolation based on objective analysis Gaussian method was performed^57,58^ for salinity (Fig. 3a), water temperature (Fig. 3b) and density (Fig. 3c) data. Seawater density was calculated using the Gibbs SeaWater (GSW) oceanographic toolbox^59^ available at http://www.teos-10.org/pubs/gsw/html/gsw_contents.html.

The buoyancy frequency $N^{2}$ was calculated using the *gsw_Nsquared.m* script available at GSW toolbox^59^.

### Satellite imagery

The remote sensing imagery used in this work consists of enhanced quasi true-color composites from Sentinel-2 MSI sensor with a 10 m spatial resolution. This study contains Copernicus Sentinel-2 2017 data processed at level 2A by CNES for THEIA Land data centre, which are freely available at https://www.theia-land.fr/. The Theia website provides Sentinel-2 images corrected for atmospheric effects and ortho-rectified^60,61^. A total of 162 cloud-free optical images from both Sentinel 2-A and 2-B were selected and downloaded for a period between 22/12/2015 and 31/12/2018. After visual analysis on the Sentinel Application Platform (SNAP) version 6.0, 36 images with evidence of IWs generated by the river plume were selected for analysis. The contrast on the ocean color images was considered sharp enough to make a quantitative analysis on 18 out of 36 images.

A proxy for the energy of the IWs was defined by adapting for oceancolor products the same methodology described by New and da Silva^56^ and Magalhães and da Silva^62^ to Synthetic Aperture Radar (SAR) images. After an image resampling and a reprojection in SNAP, profiles of surface reflectance at 560 nm ($SR_{560} ,$ green channel—band 3, major plume contrast—Valente and da Silva^38^ and Mendes et al*.*^31^) were taken through the approximate center of the IWs packets and perpendicularly to the wave crests. The normalized radiance intensity (*K*) along the profile was obtained as:

$$K = \frac{{SR_{560} - I_{0} }}{{I_{0} }}$$

where $I_{0}$ is a reference background value of surface reflectance, calculated by averaging $SR_{560}$ profile using a 30-point moving average (Fig. 1d).

An amplitude, $\alpha$, and wavelength, $\lambda ,$ were determined from the image profiles (as shown in Fig. 1d), and both parameters were used to calculate an energy proxy for each IW packet^62^. We note that, here, *λ* is the distance between consecutive IWs (and hence a measure of their wavelength), and that $\alpha$ is not to be taken as a real amplitude but rather a measure of the waves’ ‘strength’ since large amplitudes are expected to have stronger surface signals and vice-versa. Following from previous works^62–64^, the energy proxy here was calculated per unit crestlength ($E^{*}$) and is defined as:

$$E^{*} = \mathop \sum \limits_{i = 1}^{n} \alpha_{i}^{2} \lambda_{i}$$

where $n$ is the number of individual waves in the packet and the indexation variable ($i$) refers to the $i$ th wave in the packet. Note that this quantity ($E^{*}$) should not be identified with the physical energy in a wave packet but serves as a proxy measure of their strength, meant for comparisons between different packets or even in different studies^62,63^.

The IW amplitude derived from the satellite imagery, $a_{sat} ,$. was calculated based on the identification method using SAR images in a KdV wave model^65,66^. We adapted the method assuming the green surface reflectance peaks in the profile correspond, approximately, to the surface rips in the SAR images (convergence zones at the surface). For the waveform, the characteristic scale is the wave half-width ($L$) which can be directly evaluated from the image intensity profile by the distance between a consecutive peak and trough, $D$, associated with the IW. The relationship between $D$ and $L$ is given simply by $L = D/1.32$. The $a_{sat}$ (maximum amplitude) is defined by:

$$a_{sat} = \frac{12\beta }{{\alpha L^{2} }}$$

where $\beta$ and $\alpha$ are the non-linear and dispersive coefficients^65^ can be defined as $\alpha = \left[ {3\left( {h_{1} - \left( {H - h_{1} } \right)} \right)/2\left( {h_{1} \left( {H - h_{1} } \right)} \right)} \right]c_{0}$, and $\beta = \left( {h_{1} \left( {H - h_{1} } \right)/6} \right)c_{0}$.

### Model

The simulations were carried out using the 2-D nonhydrostatic model presented by Bourgault and Kelley^67^ and share a similar initially configuration with the simulations presented in Bourgault et al*.*^26^ The density and velocity profiles are constructed using an hyperbolic tangent function based on observations (Fig. 2d).

The highest horizontal resolution, Δ*x* = 0.5 m in the [− 500 m, 500 m] interval, and the resolution exponentially decreases to Δ*x* = 1 km outside the central domain of interest. The vertical resolution is set to Δ*z* = 0.1 m between the surface and 10 m depth, and similarly to Δ*x*, it decreases to reach Δ*z* = 0.5 m at a depth of 15 m.

Other simulations were carried out to test the sensitivity of the results of runs 1, 2, and 3 by decreasing the vertical resolution by a factor of 3 for the mid-resolution, and 5 for the low resolution simulations. The main features are well represented in all test simulations, although those appear more diffuse in lower resolution simulation, in line with Bourgault et al*.*^26^ results. Quantitatively, the largest amplitude leading wave produced by the low-resolution run differs by 2 cm and it is constant regardless of the plume velocity, which corresponds to a difference of about 10% considering Run#3. These values are cut by half in mid resolution simulations. The same results pattern is observed in the front velocity. The differences between high and low-resolution simulations are about 0.04 m s^−1^, however, no significant changes were observed in the leading wave velocity. The main conclusions drawn from these simulations are therefore considered to be insensitive to grid resolution.

The depth-integrated perturbation energy density flux radiating away from the front in both directions at $x = -$ 500 m (‘−’ subscript) and $x = +$ 500 m (‘+’ subscript) is calculated as described in Lamb and Nguyen^68^, Zhang et al*.*^4^ and Bourgault et al*.*^26^.

The square of the shears at the pycnocline and buoyancy frequencies on ambient (A) and plume (P) sides are, respectively,$S_{A,P}^{2} = \left[ {\left( {u_{1,2} - u_{01,02} } \right)/2d_{1,2} } \right]^{2}$ and $N_{A,P}^{2} = - \left( {g/\rho_{0} } \right)\left( {\rho_{1,2} - \rho_{0} } \right)/2d_{1,2}$. The minimum Richardson and the composite Froude numbers are respectively calculated by $Ri_{A,P} = N_{A,P}^{2} /S_{A,P}^{2}$ and $Fr_{A,P} = \sqrt {u_{1,2}^{2} /\left( {g_{1,2}^{^{\prime}} h_{1,2} } \right) + u_{01,02}^{2} /\left[ {g_{1,2}^{^{\prime}} \left( {H - h_{1,2} } \right)} \right]}$, where $g_{1,2}^{^{\prime}} = 2d_{1,2} N_{A,P}^{2}$ is the reduced gravity. The degree of non-equilibrium can be characterized by the non-dimensional density ratio, $\varepsilon = \left[ {g_{AP}^{^{\prime}} h_{2} - g_{01}^{^{\prime}} \left( {h_{1} - h_{2} } \right)} \right]/g_{01}^{^{\prime}} h_{1}$, where $g_{AP}^{^{\prime}} = g\left[ {\left( {\rho_{1,2} - \rho_{2,1} } \right)/\rho_{0} } \right]$. The results for these parameters are listed in Table 2.

Relative vorticity, $\zeta$, was calculated based on motion $V = \left( {u,w} \right)$, which in this particular 2D case, represent the $y$ component, $\zeta \left( {x,z} \right) = \frac{\partial w}{{\partial x}} - \frac{\partial u}{{\partial z}}$. The horizontal divergence is express by $div\left( V \right) = \frac{\partial u}{{\partial x}} + \frac{\partial w}{{\partial z}}$. Positive values mean a fluid expansion (divergence) whereas negative values imply convergence.

## Supplementary Information

Supplementary Information.

## Data Availability

SAR imagery is available from DLR projects OCE3154 and OCE2254 and can be provided upon request. The numerical modeling data used in this manuscript are available on request. Sentinel 2 imagery processed at level 2 is available from https://www.theia-land.fr/.
