# Author Correction: On the generation of internal waves by river plumes in subcritical initial conditions

**DOI:** 10.1038/s41598-023-38503-0

**Published:** 2023-07-13

**Authors:** R. Mendes, J. C. B. da Silva, J. M. Magalhaes, B. St-Denis, D. Bourgault, J. Pinto, J. M. Dias

**Affiliations:** 1 CIIMAR - Interdisciplinary Centre of Marine and Environmental Research, University of Porto, Matosinhos, Portugal; 2 CESAM - Centre for Environmental and Marine Studies, Physics Department, University of Aveiro, Campus de Santiago, 3810-193 Aveiro, Portugal; 3 Department of Geoscience, Environment and Spatial Planning (DGAOT), Faculty of Sciences, University of Porto, Rua Do Campo Alegre, 687, 4169-007 Porto, Portugal; 4 Institut de Sciences de La Mer de Rimouski, Université du Québec À Rimouski, 310 allée des Ursulines, Rimouski, QC G5L 3A1 Canada; 5 LSTS - Underwater Systems and Technology Laboratory, Department of Electrical and Computer Engineering, School of Engineering University of Porto, University of Porto, 4200-465 Porto, Portugal; 6 Instituto de Ciências da Terra, Polo Porto, Universidade do Porto, Rua do Campo Alegre 687, 4169-007 Porto, Portugal

Correction to: *Scientific Reports* 10.1038/s41598-021-81464-5, published online 21 January 2021

The original version of this Article contained an error in the Acknowledgements section, where the grant number of the DORIS project was incorrect.

“This work was partially funded under the project DORIS, PTDC/EEI-AUT/32335-POCI-01-0145-FEDER-032335—funded by FEDER funds through COMPETE2020—Programa Operacional Competitividade e Internacionalização (POCI) and by national funds (PIDDAC) through FCT/MCTES and under the project ”ENDURANCE—Sistema baseado em veículos autónomos para observação oceanográfica de longa duração”, supported by Norte Portugal Regional Operational Programme (NORTE2020), under the PORTUGAL 2020 Partnership Agreement, through the European Regional Development Fund (ERDF).”

now reads:

“This work was partially funded under the project DORIS, PTDC/EAM-OCE/32325/2017-POCI-01-0145-FEDER-032325—funded by FEDER funds through COMPETE2020—Programa Operacional Competitividade e Internacionalização (POCI) and by national funds (PIDDAC) through FCT/MCTES and under the project ”ENDURANCE—Sistema baseado em veículos autónomos para observação oceanográfica de longa duração”, supported by Norte Portugal Regional Operational Programme (NORTE2020), under the PORTUGAL 2020 Partnership Agreement, through the European Regional Development Fund (ERDF).”

The original Article has been corrected.

