# Supplementary material for: On the generation of internal waves by river plumes in subcritical initial conditions
Source: Sci Rep. 2021 Jan 21;11:1963. doi: 10.1038/s41598-021-81464-5 (PMC7820228; doi:10.1038/s41598-021-81464-5)
Supplement: Supplementary file 1 — Supplementary Information. [file 41598_2021_81464_MOESM1_ESM.zip › Supplementary_Material_description.docx]

**On the generation of internal waves by river plumes in subcritical initial conditions**

**Mendes, R. ^1,2^, da Silva, J.C.B ^1,3^, Magalhães, J.M. ^1,3^, St-Denis, B. ^4^, Bourgault, D. ^4^, José Pinto ^5^, João M. Dias ^2^.**

^1^ CIIMAR - Interdisciplinary Centre of Marine and Environmental Research, University of Porto, Matosinhos, Portugal

^2^ CESAM - Centre for Environmental and Marine Studies, Physics Department, University of Aveiro, Campus de Santiago, 3810-193 Aveiro, Portugal

^3^ Department of Geoscience, Environment and Spatial Planning (DGAOT), Faculty of Sciences University of Porto, Rua do Campo Alegre, 687, 4169-007 Porto, Portugal

^4^ Institut de sciences de la mer de Rimouski, Université du Québec à Rimouski, 310 allée des Ursulines, Rimouski, Québec, G5L 3A1, Canada

^5^ LSTS - Underwater Systems and Technology Laboratory, Dept. of Electrical and Computer Engineering, School of Engineering University of Porto, University of Porto, 4200-465 Porto, Portugal

**Supplementary Material**

**SM1.png**

**The relation between the location of IW and the distance to the river mouth**. Scatter plot between the maxima distance of IW manifestation in satellite observation and river mouth, and time after the low-tide in hours, showing the river discharge conditions for each observation.

**Run1_hres.mp4**

**Run#1 Video**. Visualization of the full numerical simulation from Run#1.

**Run2_hres.mp4**

**Run#2 Video**. Visualization of the full numerical simulation from Run#2.

**Run3_hres.mp4**

**Run#3 Video**. Visualization of the full numerical simulation from Run#3.

**Run4_hres.mp4**

**Run#4 Video**. Visualization of the full numerical simulation from Run#4.

**Run5_hres.mp4**

**Run#5 Video**. Visualization of the full numerical simulation from Run#5.

**Run6_hres.mp4**

**Run#6 Video**. Visualization of the full numerical simulation from Run#6.

**Run7_hres.mp4**

**Run#7 Video**. Visualization of the full numerical simulation from Run#7.
