# Supplementary figures and images for: On the generation of internal waves by river plumes in subcritical initial conditions
Source: Sci Rep. 2021 Jan 21;11:1963. doi: 10.1038/s41598-021-81464-5 (PMC7820228; doi:10.1038/s41598-021-81464-5)

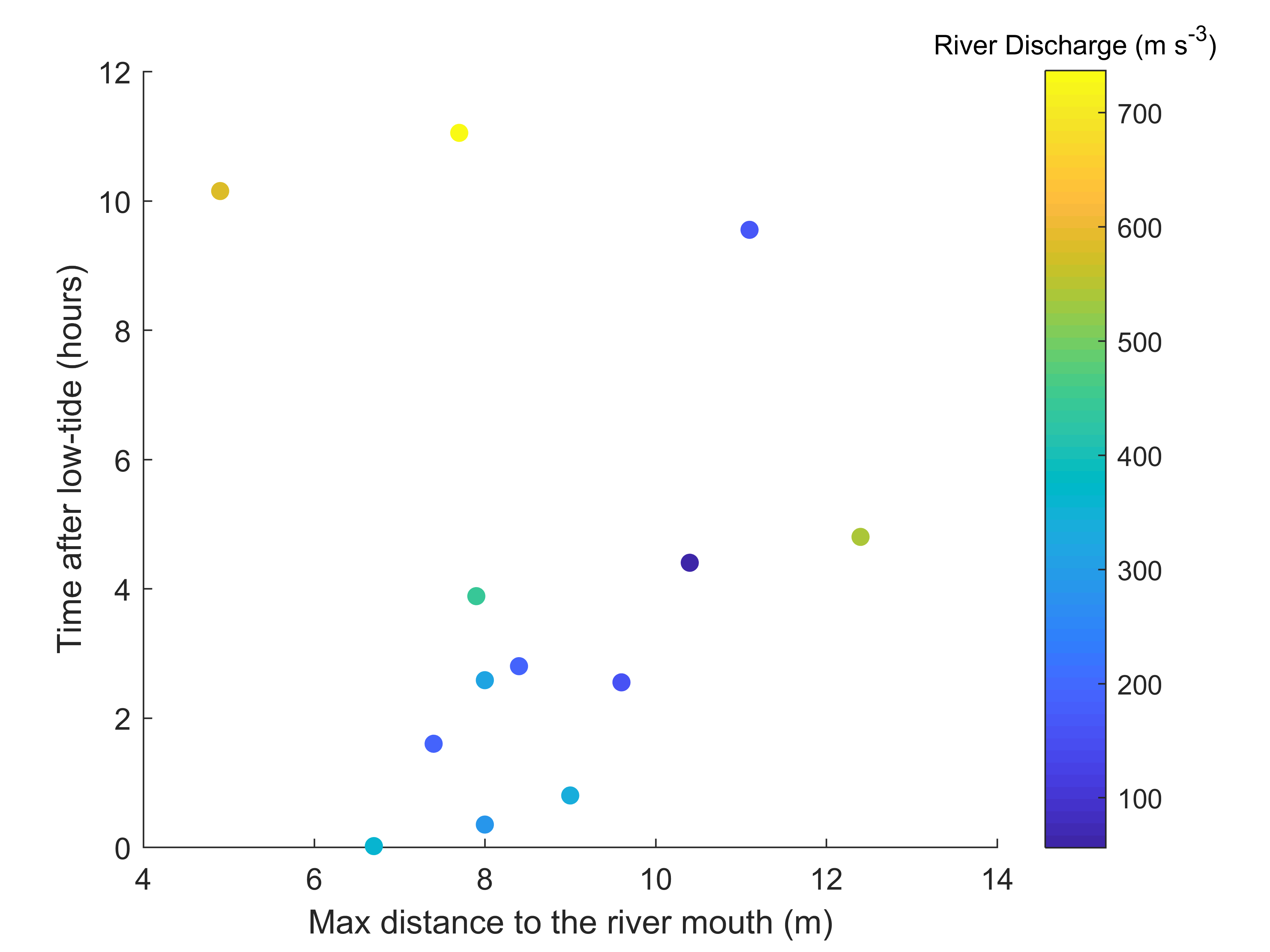

Supplement: Supplementary file 1 — Supplementary Information. [file 41598_2021_81464_MOESM1_ESM.zip › SM1.png]
